# The Role of Anti-Interferon-α Autoantibodies in Severe COVID-19: Implications for Vaccination Prioritization

**DOI:** 10.3390/vaccines13070742

**Published:** 2025-07-09

**Authors:** Xin Rong Lim, Shiyu Liu, Hwee Siew Howe, Khai Pang Leong, Elampirai Elangovan, Chiung-Hui Huang, Kok Ooi Kong, Bernard Yu Hor Thong, Shawn Vasoo, Bernard Pui Lam Leung

**Affiliations:** 1 Department of Rheumatology, Allergy and Immunology, Tan Tock Seng Hospital, Singapore 308433, Singapore; 2 National Centre for Infectious Disease, Tan Tock Seng Hospital, Singapore 308442, Singapore; 3 Department of Paediatrics, Yong Loo Lin School of Medicine, National University of Singapore, Singapore 117549, Singapore; 4 Health and Social Sciences, Singapore Institute of Technology, Singapore 828608, Singapore

**Keywords:** IFN-α, type I interferon, autoantibody, SARS-CoV-2, COVID-19 vaccination

## Abstract

**Background/Objectives**: Neutralizing autoantibodies against type I interferons, particularly interferon-alpha (IFN-α), have been implicated in severe COVID-19 outcomes. This study investigated the prevalence and functional significance of anti-IFN-α autoantibodies (AAbs) in hospitalized unvaccinated COVID-19 patients and their association with COVID-19 disease severity. **Methods**: We retrospectively analyzed serum samples from 122 hospitalized COVID-19 patients (asymptomatic/mild: n = 69, moderate: n = 35, severe/critical: n = 18) and 32 healthy uninfected controls. Anti-IFN-α AAbs were quantified using a commercial enzyme-linked immunosorbent assay (ELISA) kit, with functional neutralization assessed via competitive ELISA and STAT1 phosphorylation inhibition. Statistical comparisons were performed using one-way ANOVA for parametric data and the Kruskal–Wallis test for non-parametric variables. **Results**: Anti-IFN-α AAbs were detected in 24.6% of COVID-19 patients, with all clinical subgroups showing significantly higher titers compared to healthy controls (*p* < 0.05). Although no significant differences in anti-IFN-α AAb levels were found between mild, moderate, and severe cases, patients with severe or critical COVID-19 had markedly higher mean titers (10,511.3 ng/mL) compared to non-severe (mild + moderate) cases (375.2 ng/mL, *p* < 0.001). Strongly neutralizing anti-IFN-α AAbs, with high titers (>20,000 ng/mL) and the ability to inhibit STAT1 phosphorylation, were identified in three severe COVID-19 cases. Anti-IFN-α AAb levels correlated positively with CRP (r = 0.80, *p* < 0.0001), LDH (r = 0.80, *p* = 0.001), and neutrophil count (r = 0.52, *p* = 0.003), and negatively with lymphocyte count (r = −0.59, *p* = 0.0006). **Conclusions**: Elevated and functionally neutralizing anti-IFN-α AAbs were associated with severe COVID-19. These findings support their role as a risk factor for poor outcomes and emphasize the importance of early COVID-19 vaccination. Screening may help identify high-risk individuals, particularly those unvaccinated or with immune vulnerabilities.

## 1. Introduction

The coronavirus 2019 (COVID-19) pandemic has sparked interest in the immune system’s role in viral infections. Type I interferons (IFNs), including IFN-α, IFN-β, IFN-ε, IFN-κ, and IFN-ω, are produced by infected cells as an early response to limit viral replication. These IFNs signal through their receptors, IFNAR1 and IFNAR2, to activate the Janus kinase/signal transducers and activators of transcription (JAK/STAT) pathway, leading to the induction of IFN-stimulated genes (ISGs) that mediate antiviral, antiproliferative, and adaptive immune responses [1].

Defective type I IFN host response through impaired IFN signaling or production from underlying factors, including genetic and autoimmune phenomena, allows for continued viral replication, impairing the host’s ability to effectively combat the virus [2]. The COVID Human Genetic Effort led by Zhang Q and colleagues identified mutations in key pathways involved in type I IFN immunity, such as the Toll-like receptor 3 (TLR3) and interferon regulatory factor 7 (IRF7) pathways, in patients with life-threatening COVID-19 pneumonia [3]. These mutations were seen in individuals who had no prior history of severe infections. Autoantibodies directed against IFN-α, a key member of the type I IFN family, represent a form of acquired immune dysregulation that directly impairs antiviral responses. Bastard et al. demonstrated that neutralizing autoantibodies against type I interferons can abrogate IFN-mediated signaling, effectively disabling the host’s first line of antiviral defense. These autoantibodies were present in 10.2% of patients with life-threatening COVID-19 [4]. The majority of these patients were men, and the presence of autoantibodies was associated with more severe disease outcomes [4]. Beyond type I interferon autoantibodies, various other autoantibodies have been reported in patients following SARS-CoV-2 infection, including those targeting annexin A2, angiotensin-converting enzyme 2 (ACE2), phospholipids, nuclear antigens, and other tissue-specific proteins, highlighting the broad autoimmune activation triggered by COVID-19 [5,6,7].

While anti-IFN-α autoantibodies (AAbs) may play a significant role in COVID-19 severity, their impact in the Asia Pacific region requires further study. Singapore has reported over 3 million cases and more than 2000 deaths as of 13 April 2024 [8]. Globally, the number of weekly reported COVID-19 deaths has continued to decline substantially since the peak in early 2021, reflecting the ongoing impact of widespread vaccination, improved clinical management, and population-level immunity [9]. Understanding the role of anti-IFN-α AAbs is essential for optimizing vaccination approaches and improving COVID-19 disease management, particularly in unvaccinated individuals. This study aimed to determine the prevalence of anti-IFN-α AAbs in unvaccinated hospitalized COVID-19 patients and investigate their relationship with disease severity, as measured by the World Health Organization (WHO) clinical management of COVID-19 guidelines [10].

## 2. Materials and Methods

### 2.1. Study Design and Participants

A total of 122 hospitalized patients with confirmed COVID-19, diagnosed via nasopharyngeal swab SARS-CoV-2 real-time reverse transcriptase–polymerase chain reaction (RT-PCR), were enrolled in this study at the National Centre for Infectious Diseases (NCID) in Singapore between 28 March and 30 September 2020. This study was conducted during the early phase of the pandemic, when the original SARS-CoV-2 strain was dominant, and preceding the availability of the Pfizer BNT162b2 COVID-19 vaccine. The patients’ demographic information, comorbidities, and laboratory results were collected from their electronic medical records corresponding to the time of blood sample collection. Lymphocyte, neutrophil, and platelet counts were obtained from complete blood count (CBC) panels performed during routine clinical care; C-reactive protein (CRP) and lactate dehydrogenase (LDH) levels were measured via hospital laboratory biochemical assays at the time of serum collection. Chest X-ray (CXR) findings were interpreted by radiologists and documented in the electronic medical records at the time of hospital admission, within ±5 days of serum collection. The Charlson Comorbidity Index (CCI) was calculated for each patient to assess baseline comorbidity burden using a validated scoring system that predicts 10-year mortality based on the presence of various comorbid conditions [11]. CCI scores were extracted from a retrospective chart review at the time of COVID-19 diagnosis. The severity of COVID-19 was defined according to the World Health Organization (WHO) criteria based on clinical record charting and chest imaging [10], as shown below.
World Health Organization case definition of COVID-19 disease severity**Asymptomatic/Presymptomatic****Test Positive for SARS-CoV-2 with a Virologic Test but Have No Symptoms Consistent with COVID-19**MildIndividuals showing any signs/symptoms of COVID-19 (e.g., fever, cough, sore throat, malaise, headache, myalgia, nausea, vomiting, diarrhea, loss of taste/smell) but who do not have shortness of breath or clinical signs of pneumonia or abnormal chest imagingModerateIndividuals showing evidence of lower respiratory tract disease during clinical assessment or imaging and who have a SpO2 ≥ 94% on room airSevereIndividuals who have a SpO2 of <94% on room air or a P/F ratio of <300 mmHg, a respiratory rate of >30 breaths/min, or lung infiltrates occupying >50% of lung fieldsCriticalIndividuals with respiratory failure, septic shock, and/or multiple organ dysfunction

Immunological assays were conducted on leftover frozen serum samples collected as part of standard clinical evaluation, with a waiver of consent approved by the institutional review board (DSRB 2020/00910 and DSRB 2020/00741). Samples were collected at a mean of 8.61 ± 8.63 days following symptom onset. Frozen serum samples from 32 healthy, uninfected individuals, obtained prior to the COVID-19 pandemic, were used as controls to establish baseline anti-IFN-α AAb levels in the uninfected population.

### 2.2. Enzyme-Linked Immunosorbent Assay (ELISA) for the Detection of Anti-IFN Alpha (Anti-IFN-α) Autoantibodies and Neutralization Assay

Anti-IFN-α AAbs were quantified by a commercial ELISA precoated with recombinant human IFN-α (BMS217, Bender MedSystems GmbH, Vienna, Austria). Serum from 32 healthy, uninfected individuals tested with the above anti-IFN-α ELISA showed low-level antibody detection in 26 cases, with a mean concentration of 14 AU/mL (±16 standard deviation).

Serum levels of anti-IFN-α AAbs were measured using the commercial ELISA kit as detailed above, following the manufacturer’s instructions. The assay’s lower limit of detection was 3.1 ng/mL, and levels exceeding 120 ng/mL were considered positive. The threshold of 120 ng/mL was determined based on the maximum value plus two standard deviations of the ELISA results from our healthy uninfected control group. Positive samples were examined using a competitive ELISA to estimate the potential neutralizing capacity, and true functional neutralization was confirmed by inhibition of IFN-α-induced signal transducers and activators of transcription STAT1 phosphorylation in T cells via flow cytometry.

To assess potential neutralizing capacity, positive anti-IFN-α AAb patients’ serum samples were serially diluted 10 folds (10^2^ to 10^6^) in PBS/10% fetal bovine serum assay buffer spiked with 200 pg/mL of recombinant IFN-α (ThermoFisher Scientific, Waltham, MA, USA) in a shaking incubator for 1 h at room temperature before being assayed by IFN-α ELISA (Bender MedSystems GmbH). In the competitive ELISA assay, the blocking index was defined as the maximum dilution from (10^2^ to 10^6^) half-maximal inhibitory concentration (IC_50_).

### 2.3. Functional Evaluation of Anti-IFN-α Autoantibodies

Peripheral blood mononuclear cells (PBMCs) were stimulated in vitro with recombinant human IFN-α (400 ng/mL, e-Bioscience), either alone or in the presence of patient serum at serial dilutions (1%, 3%, and 10%) for 30 min at 37 °C. Cells were fixed in 2% paraformaldehyde/PBS (10 min at 37 °C) and then permeabilized in BD Phosflow™ Perm Buffer III and stained with PE-conjugated phospho-STAT1 antibodies. The extent of STAT1 phosphorylation was quantified by flow cytometry according to the BD Phosflow protocol (BD Biosciences, San Diego, CA, USA) and performed as part of the clinical immunology diagnostic workflow for patient assessment (National University Health System, Singapore; personal communication to Lim XR, as indicated in Supplementary Figure S1).

### 2.4. Statistical Method

Patient characteristics were summarized using descriptive analyses. All statistical analyses were performed using the Intercooled STATA (Stata Corporation, College Station, TX, USA). Continuous variables were expressed as medians (interquartile ranges, IQRs) or means (standard deviations, SDs); one-way ANOVA was used for parametric data and the Kruskal–Wallis test for non-parametric variables, followed by pairwise Mann–Whitney U tests for multiple comparisons. Correlations of levels of anti-IFN-α AAbs with levels of CRP, LDH, platelets, lymphocytes, and neutrophils were assessed using Spearman correlation.

## 3. Results

Of the 122 patients, 87 (71.3%) were male, with a mean age of 45 years. The cohort was diverse, with patients of Chinese (34.4%), Malay (13.1%), Indian/Bangladeshi (40.2%), and other ethnicities (12.3%). A total of 18 (14.8%) suffered from severe COVID-19, 35 (28.7%) had moderate COVID-19, and 69 (56.6%) were asymptomatic/mild COVID-19 cases. Patient blood samples were collected at a mean of 8.61 ± 8.63 days following symptom onset.

Patients with severe/critical COVID-19 were generally older (mean age = 54.83 years) compared to those with moderate (50 years) and mild/asymptomatic disease (40 years) (*p* < 0.001). They also had a higher prevalence of elevated BMI > 25 (33.3%) compared to moderate (17.1%) and mild/asymptomatic patients (10.1%), although this did not reach statistical significance (*p* = 0.052). The Charlson Comorbidity Index was highest in the severe/critical group (mean = 1.89), followed by moderate (1.34) and mild/asymptomatic (0.59) groups (*p* < 0.001). Markers of inflammation, including CRP and LDH, were significantly elevated in severe/critical patients (mean CRP = 115.23 mg/L; LDH = 812.06 U/L) compared to moderate (CRP = 35.72 mg/L; LDH = 457.9 U/L) and mild/asymptomatic groups (CRP = 7.67 mg/L; LDH = 372.6 U/L) (both *p* < 0.001). Neutrophil counts were also significantly higher in the severe/critical group (mean = 6.86 × 10^9^/L) compared to the moderate (3.74 × 10^9^/L) and mild (3.71 × 10^9^) groups (*p* < 0.001), while lymphocyte counts showed no significant difference across groups (*p* = 0.433) (Table 1).

**Figure 1:**
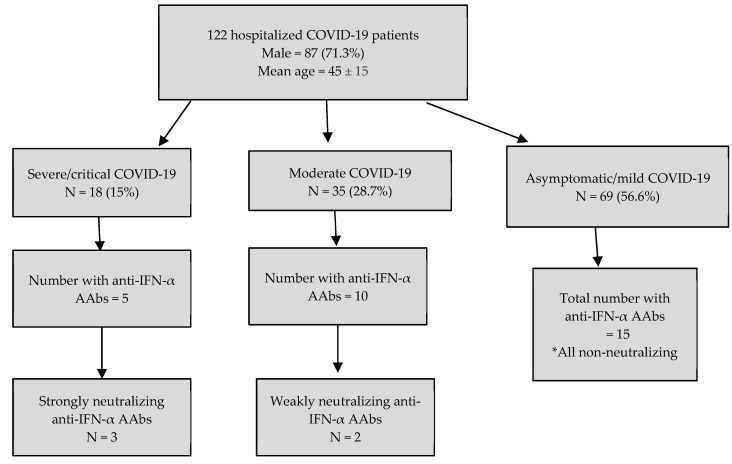
Proportion of anti-IFN-α AAbs in hospitalized Singaporean COVID-19 patients.

A total of 24.6% (n = 30) of patients tested positive for anti-IFN-α AAbs based on the cut-off of >120 ng/mL via commercial ELISA, and 5 (4.1%) of them exhibited neutralizing activity, with an IC_50_ determined by competitive binding ELISA ranging from 1:100 to 1:68,000 (Supplementary Table S1). Anti-IFN-α AAbs were found in 27.8% of severe/critical COVID-19 patients, compared to 28.6% of moderate cases and 21.7% of mild/asymptomatic cases (Figure 1).

Three patients in the severe COVID-19 group demonstrated strongly neutralizing anti-IFN-α AAbs, with titers exceeding 20,000 ng/mL, IC_50_ values ranging from 5000 to 68,000, and inhibition of IFN-α-induced STAT1 phosphorylation as assessed by flow cytometry. In the moderate COVID-19 group, two patients had weakly neutralizing anti-IFN-α autoantibodies, with IC_50_ values between 100 and 350; however, these antibodies did not inhibit STAT1 phosphorylation. Detailed data on these findings are provided in Supplementary Table S1. In contrast, no neutralizing activity was observed in the asymptomatic/mild COVID-19 group.

All three patients with strongly neutralizing anti-IFN-α AAbs were Chinese and aged between 69 and 70 years old and comprised two males and one female. All had a Charlson Comorbidity Index of 4 and experienced severe/critical COVID-19 necessitating admission to the intensive care unit, though only one patient required mechanical ventilation. One patient was treated with lopinavir/ritonavir, another received both lopinavir/ritonavir and IFN-β, while the third patient unfortunately died, despite receiving tocilizumab and convalescent plasma from recovered COVID-19 patients. The outcomes for the first two patients remain unclear since only cross-sectional data were available.

**Figure 2:**
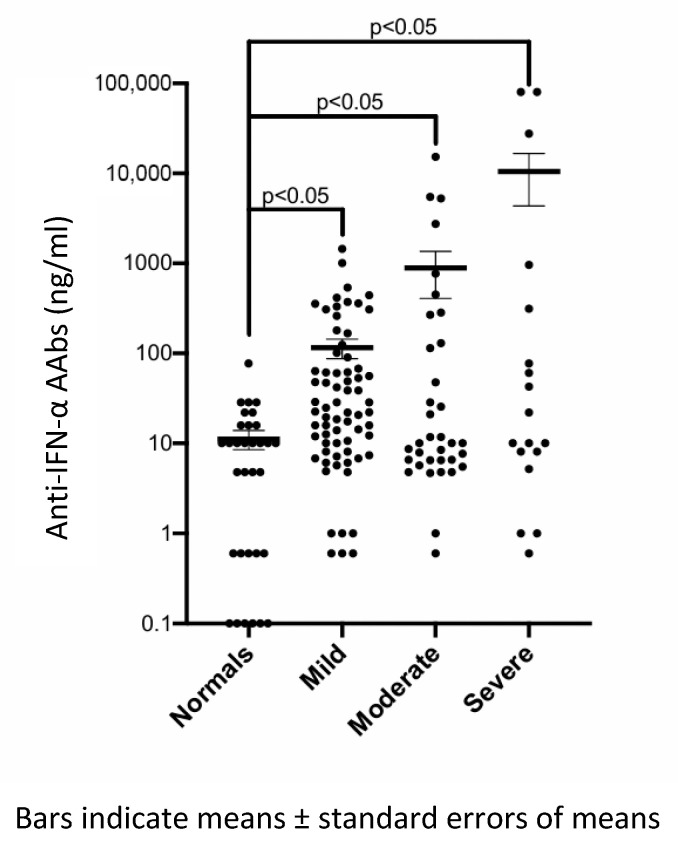
Scatterplot comparing anti-IFN-α autoantibody (AAb) titers across four groups: normal controls and mild, moderate, and severe COVID-19 cases.

Severe COVID-19 patients had significantly higher mean titers of anti-IFN-α AAbs (10,511.3 ng/mL) compared with non-severe (mild + moderate) patients (375.2 ng/mL) (*p* < 0.001). A scatter plot compared anti-IFN-α AAb titers across the four groups: healthy controls and patients with mild, moderate, and severe/critical COVID-19 are shown in Figure 2. Statistical analysis using the Mann–Whitney U test demonstrated significantly higher antibody titers in all COVID-19 subgroups compared to healthy controls (*p* < 0.05 for each comparison). However, pairwise comparisons between COVID-19 severity subgroups (mild vs. moderate, mild vs. severe, and moderate vs. severe) did not reach statistical significance.

We observed that anti-IFN-α AAb levels correlated positively with CRP (r = 0.80, *p* < 0.0001), LDH (r = 0.80, *p* = 0.001), and neutrophil count (r = 0.52, *p* = 0.003), and negatively with lymphocyte count (r = −0.59, *p* = 0.0006) (Table 2).

## 4. Discussion

The prevalence of anti-IFN-α AAbs in our cohort (24.6%) aligns with emerging global data. A systematic review reported prevalence rates of circulating autoantibodies against IFN-α and IFN-ω at 7.2% and 4.4%, respectively, across patient populations irrespective of disease severity [12]. Notably, 16.7% of patients in our study with severe or critical COVID-19 exhibited functionally strong neutralizing anti-IFN-α AAbs comparable to the 9–11% prevalence observed in severe cohorts from Spain and Shanghai [13,14,15,16]. Despite the presence of anti-IFN-α AAbs in 24.6% of patients in our cohort, only a minority (4.1%) exhibited strong neutralization activity. This highlights the importance of evaluating not just the presence of autoantibodies, but also their functional activity. Functional assays that determine neutralizing potency are critical for identifying patients at genuine risk of clinical deterioration.

**Figure 3:**
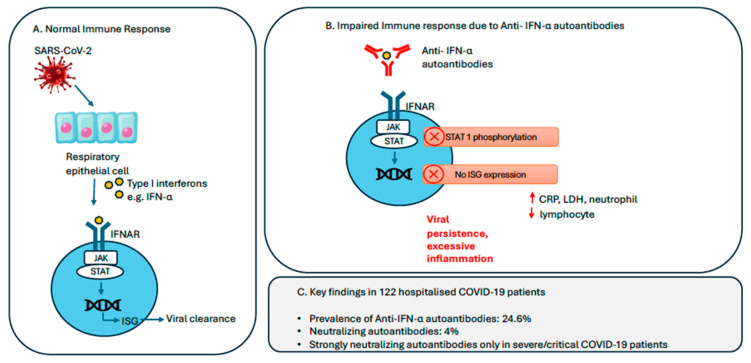
Lmpact of anti-IFN-α autoantibodies on COVID-19 severity. (**A**) In a normal immune response, SARS-CoV-2 infection activates respiratory epithelial cells to produce type I interferons (e.g., IFN-α) which bind to IFNAR, triggering the JAK-STAT signaling cascade and upregulation of ISGs that promote viral clearance. (**B**) In contrast, anti-IFN-α autoantibodies block this pathway, impairing ISG activation and resulting in viral persistence and excessive inflammation. (**C**) Among 122 hospitalized COVID-19 patients, anti-IFN-α autoantibodies were detected in 24.6%, with 4% harboring neutralizing activity. Strongly neutralizing antibodies were exclusively observed in patients with severe/critical COVID-19 infection. Abbreviations: IFNAR, Type I IFN receptor; ISC, IFN-stimulated gene; JAK, Janus kinase; STAT, signal transducer and activator of transcription.

Our data provide compelling evidence that these neutralizing AAbs are clinically significant, as they were detected predominantly in patients with severe or critical illness. These patients exhibited markedly higher serum titers (mean = 10,511.3 ng/mL), robust neutralization activity (IC_50_ up to 1:68,000), and impaired STAT1 phosphorylation upon IFN-α stimulation, confirming functional disruption of the interferon pathway. In contrast, mild/asymptomatic patients lacked neutralizing activity, even if they had detectable antibody levels. These neutralizing autoantibodies disrupt type I interferon signaling, a crucial component of early antiviral defense, by inhibiting STAT1 phosphorylation and the downstream induction of interferon-stimulated genes (ISGs) (Figure 3). Type I IFNs are pivotal in bridging innate and adaptive immunity, initiating early viral clearance and shaping downstream T and B cell responses. We postulate that the presence of neutralizing anti-type I interferon autoantibodies impairs early innate immune responses, allowing uncontrolled viral replication and delayed clearance. This defective antiviral defense likely contributes to a cascade of hyperinflammation, consistent with clinical manifestations such as acute respiratory distress syndrome (ARDS), multiorgan dysfunction, and elevated inflammatory markers observed in severe COVID-19 [17]. There is no universally standardized definition of cytokine storm; it is generally characterized by hyperinflammation, elevated circulating cytokines, and multiorgan dysfunction. In our cohort, three patients in the severe/critical COVID-19 group were clinically assessed to have features consistent with a cytokine storm and were treated with corticosteroids, with or without tocilizumab, in line with institutional protocols.

The correlation between high levels of anti-IFN-α AAbs and inflammatory markers such as CRP (r = 0.80), LDH (r = 0.80), and neutrophil counts (r = 0.52), and their inverse relationship with lymphocyte counts (r = −0.59), reflect the inflammatory dysregulation commonly seen in cytokine storm syndromes. These associations support a mechanistic link between IFN-α autoantibodies and the clinical deterioration seen in severe COVID-19 [16,18]. Among type I interferons, antibodies against IFN-α appear to have the most significant association with severe disease outcomes. For example, Bastard et al. reported a strikingly higher odds ratio (OR = 67.6) for life-threatening COVID-19 pneumonia in individuals with neutralizing antibodies against IFN-α2, compared to those with antibodies against IFN-ω (OR = 2.6) [19]. Similarly, Wang et al. found that anti-IFN-α autoantibodies were more common in severe cases (89%) than anti-IFN-ω (77%) [20].

Previous studies have reported a higher prevalence of neutralizing anti-IFN-α autoantibodies in older males with underlying comorbidities, often associated with severe or life-threatening COVID-19 outcomes [4,21]. In our cohort, all three patients with high-titer, strongly neutralizing anti-IFN-α AAbs were elderly and had multiple comorbid conditions (Charlson Comorbidity Index = 4). Two of the three were male. One patient died despite aggressive treatment, including tocilizumab and convalescent plasma. The underlying mechanisms remain unclear but may involve sex-based differences in immune regulation and predisposition to autoimmunity [22,23].

These findings have direct clinical implications:

First, they emphasize the potential utility of screening for anti-IFN-α AAbs in patients at high risk of deterioration, such as older adults or those with early signs of severe disease. The incorporation of anti-IFN-α AAb screening, particularly functional neutralization assays, into early diagnostic algorithms for COVID-19 may enable clinicians to anticipate disease trajectories, escalate monitoring, and guide the early use of immunomodulators. This is particularly pertinent in resource-limited settings, where early identification of high-risk patients can significantly impact ICU admission and ventilator allocation.

Second, while therapeutic IFN-β-1b has shown inconsistent benefits in prior trials, this may reflect suboptimal timing, patient selection, or the dominance of other pathways once cytokine dysregulation has set in [13]. Further studies should explore immune-modulating strategies for those with antibodies to type I IFNs. In patients with disseminated non-tuberculous mycobacterial infections driven by anti-IFN-γ autoantibodies, B cell-depleting therapy with rituximab has demonstrated efficacy in lowering autoantibody titers and enhancing infection control [24]. These observations support the potential utility of B cell-targeted interventions in managing diseases mediated by anti-cytokine autoantibodies. Understanding the origin or mechanisms that lead to the production of these autoantibodies will be essential for developing rational, targeted therapies.

Given that our cohort consisted primarily of unvaccinated patients, as this study was conducted in early 2020 during the pandemic, it highlights the critical protective role of COVID-19 vaccination in mitigating preexisting immune vulnerabilities. While Bastard et al. reported that some individuals with neutralizing autoantibodies to type I IFN developed severe COVID-19 despite vaccination, these cases were rare, and the vast majority still benefited from vaccination in terms of protection from critical illness [25]. Shi et al. [15] reported that a subset of vaccinated individuals with pre-existing neutralizing autoantibodies against type I interferons may be at increased risk of breakthrough hypoxemic COVID-19 pneumonia. In their study, two patients (12%) who developed life-threatening COVID-19 despite receiving two or more doses of an inactivated SARS-CoV-2 vaccine were found to have serum autoantibodies that neutralized IFN-α2 and IFN-ω at physiologically relevant concentrations. While these findings raise important considerations, the small number of cases precludes definitive conclusions about whether vaccine type, such as mRNA versus inactivated vaccines, modulates susceptibility in individuals with pre-existing anti-cytokine autoantibodies. More encouragingly, Wolff et al. showed that COVID-19 vaccination provides robust protection in individuals with autoimmune polyendocrine syndrome type I (APS-1), even those with neutralizing type I IFN AAbs [26].

Cases of yellow fever vaccine-associated adverse events (yellow fever vaccine-associated neurotropic disease or viscerotropic disease) have emerged in individuals who harbor anti-type I IFN AAbs [27]. Concerns regarding vaccine safety in individuals with anti-IFN autoantibodies are not substantiated in the context of COVID-19 vaccines. Yalcinkaya et al. demonstrated that individuals with anti-type I IFN AAbs do not experience an increased prevalence of adverse events following immunization (AEFI) with mRNA or viral vector COVID-19 vaccines [28].

### Limitations

One limitation of this retrospective study is the small sample size and cross-sectional design. The study was conducted during the early phase of the pandemic, when the original virus strain was prevalent and COVID-19 vaccination was either unavailable or not widely available. As the virus has evolved and vaccination campaigns have progressed, the findings may not fully reflect the current situation. Additionally, this study focused only on anti-IFN-α autoantibodies, while other interferons like IFN-β and IFN-ω may also play a role in disease severity.

Despite inherent limitations, this study offers a unique opportunity to characterize immunological responses in SARS-CoV-2-naïve, unvaccinated individuals. The absence of prior infection and vaccine exposure strengthens the interpretability of natural immune mechanisms in this cohort. These insights into the role of anti-IFN-α AAbs in severe COVID-19 highlight the importance of continued research into targeted therapeutic strategies such as booster vaccination to enhance B and T cell responses which may support personalized treatment approaches for individuals at higher risk of severe disease.

## 5. Conclusions

The significant prevalence of anti-IFN-α AAbs among unvaccinated hospitalized COVID-19 patients and their association with disease severity highlights the clinical importance of COVID-19 vaccination. These autoantibodies compromise early antiviral immune responses by neutralizing endogenous IFN-α, thereby facilitating unchecked viral replication and heightened inflammatory damage. While specific treatments for this immune dysfunction remain limited, COVID-19 vaccination emerges as a crucial protective measure. The findings support prioritizing vaccination, not only to prevent severe illness but also to mitigate the risks posed by undiagnosed immunological defects such as anti-IFN-α autoantibodies. Screening for anti-IFN-α AAbs could help identify high-risk individuals, particularly those who are unvaccinated or have undiagnosed immune defects.

## Figures and Tables

**Table 1:** Comparison of asymptomatic/mild/moderate and severe/critical COVID-19 patients.

Clinical Characteristics	Patients with Asymptomatic/Mild COVID-19 (N = 69)	Patients with Moderate COVID-19 (N = 35)	Patients with Severe/Critical COVID-19 (N = 18)	*p* Value
Age, years (mean ± SD)	40 ± 13.6	50 ± 13.8	54.83 ± 16.53	<0.001
Male, number (%)	49 (71%)	24 (68.6%)	14 * (77.78%)	0.779
Charlson Comorbidity Index (mean ± SD)	0.59 ± 0.92	1.34 ± 1.60	1.89 ± 1.66	<0.001
BMI > 25, number (%)	7 (10.1%)	6 (17.1%)	6 (33.33%)	0.052
Abnormal CXR (opacities/consolidation, no. (%)	0 (0%)	35 (100%)	18 (100%)	<0.001
CRP, mg/L (mean ± SD)	7.67 ± 14.76	35.72 ± 46.33	115.23 ± 94.88	<0.001
LDH, U/L (mean ± SD)	372.6 ± 78.7	457.9 ± 167.3	812.06 ± 655.28	<0.001
Lymphocyte count, 10^9^/L (mean ± SD)	1.60 ± 0.53	1.43 ± 0.70	1.36 ± 0.67	0.433
Neutrophil count, 10^9^/L (mean ± SD)	3.71 ± 1.84	3.74 ± 1.87	6.86 ± 3.65	<0.001
Platelet count, 10^9^/L (mean ± SD)	227.3 ± 73.8	219.2 ± 80.4	315.22 ± 149.71	<0.001
Anti-IFN-α AAbs, ng/mL (mean ± SD)	115.7 ± 235.3	886.6 ± 2827	10,511.3 ± 26,097	0.418

* Fourteen males, including one transgender individual; AAbs: autoantibodies; CRP: C-reactive protein; CXR: chest X-ray; LDH: lactate dehydrogenase; SD: standard deviation.

**Table 2:** Anti-IFN-α autoantibodies (AAbs) and clinical correlations.

**Anti-IFN-α AAbs and Clinical Correlations**	**Pearson Correlation Coefficient (r)**	***p* Value (*p*)**
Anti-IFN-α AAbs and CRP	0.80	<0.0001
Anti-IFN-α AAbs and LDH	0.80	0.001
Anti-IFN-α AAbs and lymphocyte count	−0.59	0.0006
Anti-IFN-α AAbs and neutrophil count	0.52	0.003
Anti-IFN-α AAbs and platelet	0.060	0.75

CRP: C-reactive protein; LDH: lactate dehydrogenase.

## Data Availability

The original contributions presented in this study are included in the article/Supplementary Materials. Further inquiries can be directed to the corresponding author(s).

## Supplementary Materials

The following supporting information can be downloaded at: https://www.mdpi.com/article/10.3390/vaccines13070742/s1, Figure S1: Signal transducer and activator of transcription 1 (STAT1) phosphorylationin normal peripheral blood CD3+ T cells after direct stimulation with recombinant human lFN-α; Table S1: Clinical phenotypes of patients with potential neutralizing capacity via competitive ELISA as well as details of the anti-IFN-α AAbs titre, competitive ELISA and STAT-1 phosphorylation.

## Abbreviations

AAb	Autoantibody
ACE2	Angiotensin-converting enzyme 2
AEFI	Adverse events following immunization
ANOVA	Analysis of variance
APS	Autoimmune polyendocrine syndrome
ARDS	Acute respiratory distress syndrome
BMI	Body mass index
CBC	Complete blood count
CCI	Charlson Comorbidity Index
CRP	C-reactive protein
COVID-19	Coronavirus disease 2019
CXR	Chest X-ray
ELISA	Enzyme-linked immunosorbent assay
IC	Inhibitory concentration
ICU	Intensive care unit
IFN	Interferon
IFNAR	Interferon alpha and beta receptor subunit
IQR	Interquartile range
IRF	Interferon regulatory factor
ISGs	Interferon-stimulated genes
JAK/STAT	Janus kinase/signal transducers and activators of transcription
LDH	Lactate dehydrogenase
mRNA	Messenger ribonucleic acid
NCID	National Centre for Infectious Diseases
OR	Odds ratio
PBS	Phosphate buffered saline
RT	Reverse transcriptase
RT-PCR	Reverse transcriptase–polymerase chain reaction
SARS-CoV-2	Severe acute respiratory syndrome coronavirus 2
SD	Standard deviation
STAT1	Signal Transducer and Activator Transcription 1
TLR	Toll-like receptor
TNF-α	Tumor necrosis factor α
WHO	World Health Organization
