# Supplementary material for: The Role of Anti-Interferon-α Autoantibodies in Severe COVID-19: Implications for Vaccination Prioritization
Source: Vaccines (Basel). 2025 Jul 9;13(7):742. doi: 10.3390/vaccines13070742 (PMC12298202; doi:10.3390/vaccines13070742)
Supplement: Supplementary file 1 [file vaccines-13-00742-s001.zip › vaccines-3637568-supplementary.pdf]

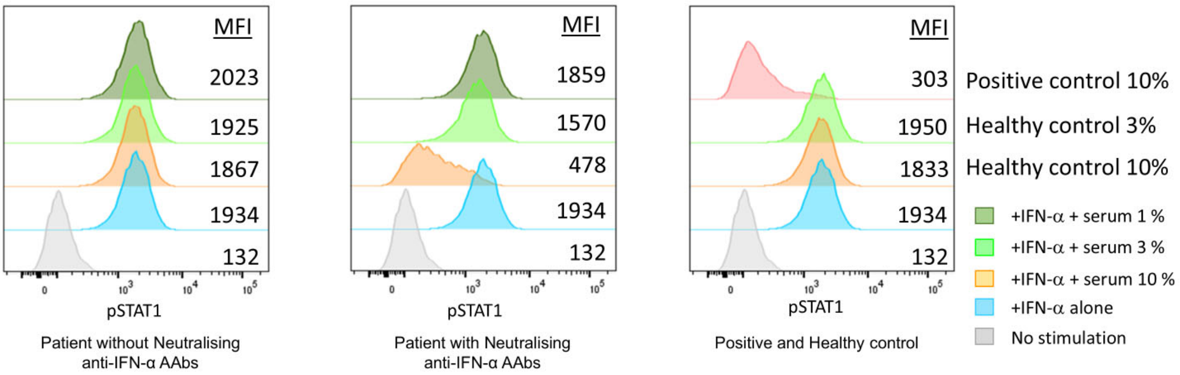

Representative results are shown in Supplementary Figure 1. Inhibition of STAT1 phosphorylation was observed in 3 samples incubated with serum from severe/critical COVID-19 patients harboring neutralizing anti-IFN-α AAbs, compared to those without such antibodies or healthy controls.

Figure S1. Signal transducer and activator of transcription 1 (STAT1) phosphorylationin normal peripheral blood $CD3^{+}$ T cells after direct stimulation with recombinant human IFN-α.

Supplementary Table S1: Clinical phenotypes of patients with potential neutralizing capacity via competitive ELISA as well as details of the anti-IFN-α AAbs titre, competitive ELISA and STAT-1 phosphorylation.

Patient	Age/ Gender	Charlson Comorbidity index	COVID-19 Severity	Anti-IFN-α AAb ELISA Titer (ng/mL)	Competitive binding ELISA, 50% inhibition at N/Dilution	Inhibition of IFN-α-induced STAT1 phosphorylation via flow cytometry	Treatment specific to COVID-19 received
1	69/M	4	Severe	>20,000	1: 68,000	Yes	Lopinavir/ritonavir
2	69/M	4	Severe	>20,000	1: 68,000	Yes	Lopinavir/ritonavir, IFN-β
3	70/F	4	Severe	>20,000	1:4759	Yes	Tocilizumab, convalescent plasma from recovered COVID-19 patients
4	59/M	1	Moderate	19312.90	1:350	No	Nil
5	72/M	1	Moderate	15263.50	1:102	No	Nil

M: male; F: female; anti-IFN-α AAbs: anti-interferon-alpha autoantibodies; IFN: interferon; ELISA: enzyme-linked immunosorbent assay; STAT1: signal transducers and activators of transcription 1
